# Assessing the Factors Associated With the Detection of Juvenile Hacking Behaviors

**DOI:** 10.3389/fpsyg.2020.00840

**Published:** 2020-05-05

**Authors:** Jin Ree Lee, Thomas J. Holt

**Affiliations:** School of Criminal Justice, College of Social Science, Michigan State University, East Lansing, MI, United States

**Keywords:** computer hacking, low self-control, social bonds, cybercrime, deterrence, juvenile delinquency

## Abstract

Research on delinquency reduction often highlights the importance of identifying and sanctioning antisocial and illegal activities so as to reduce the likelihood of future offending. The rise of digital technology complicates the process of detecting cybercrimes and technology enabled offenses, as individuals can use devices from anywhere to engage in various harmful activities that may appear benign to an observer. Despite the growth of cybercrime research, limited studies have examined the extent to which technology enabled offenses are detected, or the behavioral and attitudinal factors associated with being unobserved or caught for one’s actions. The current study addresses this gap in the literature by estimating a multinomial regression model for self-reported computer hacking behavior and the likelihood of those actions being detected in a large international sample of juveniles (*N* = 51,059). The findings demonstrate significant differences between youth who hack without detection compared to those who are caught. The implications of this analysis for our understanding of cybercrime and its relationship to traditional delinquency are explored in depth.

## Assessing the Factors Associated With the Detection of Juvenile Hacking Behaviors

For many forms of crime and delinquency, the notion of deterring behavior is imperative so as to reduce the risk of future offending. Deterrence is generally derived from the perceived threat of detection and sanctioning for wrongdoing, whether from police or informal sources of control such as peers or parents in the case of delinquency (Nagin and Pogarsky, 2001; Pratt et al., 2006). The decision to offend is thus a calculus of the perceived likelihood of detection relative to the reward acquired from the offense (Cornish and Clarke, 2014). Detection is, however, variable based on the nature of the offense and its situational characteristics, such as the presence of surveillance tools and observers to report wrongdoing (Clarke, 1997; Cornish and Clarke, 2003; Reyns, 2010). As a result, the risk of detection varies based on the extent to which offenders can obfuscate their behaviors and otherwise appear to engage in normal behaviors in physical space (Wright and Decker, 1996; Cherbonneau and Copes, 2005; Cardone and Hayes, 2012).

The rise of computers and the Internet have created new opportunities to engage in crimes that are more difficult to detect through traditional means (Yar, 2013; Holt and Bossler, 2016). Individuals can engage in so-called cybercrimes where their use of technology enables them to commit an offense from the comfort of their home without the need to interact with their victims in public settings (Holt and Bossler, 2016). In addition, parents and/or guardians who may observe offline deviant behaviors may not notice cybercriminality because the individual may simply appear to be typing on a keyboard or utilizing a specific program to access content (Holt and Bossler, 2016). Actors may also conceal illegal online activitiy by taking their laptop or electronic device into a private space so as to avoid being asked questions by family members or guardians (Holt et al., 2019).

These factors may all lower the perceived risk of detection for engaging in cybercrime, as the rate of arrest is extremely low proportionally to physical crimes (see Holt and Bossler, 2016). This is especially true for computer hacking, generally defined as the use of technological understanding to engage in unauthorized access of computer systems and networks (Jordan and Taylor, 1998; Wall, 2001; Furnell, 2002; Schell and Dodge, 2002; Holt, 2007; Holt et al., 2019). Though hacking can be used for legitimate applications, the behavior has largely been associated with malicious, criminal activity in the general public over the last two decades (Furnell, 2002; Holt, 2007; Grabosky, 2016). As a result, hacking is frequently viewed as a serious threat affecting both the public and private sector.

Research regarding hackers and hacking have increased over the last two decades, providing insight into key individual predictors for hacking among juvenile and adult samples (see Holt and Bossler, 2016 for review). Research examining the detection of hacking is nascent in the broader literature (see Maimon et al., 2014), calling to question what factors differentiate hackers from non-hackers as to their likelihood of being caught for their involvement in an increasingly common form of cybercrime. Such information is vital to better understand the factors that may increase an actor’s willingness to hack, as well as decrease their likelihood of detection. In turn, better detection and prevention strategies can be developed to curb hacking behavior among youth, regardless of their ability to conceal their actions.

The current study attempted to address this question through the use of a clustered multinomial regression model of an international sample of over 51,000 juveniles. The model compared those who hacked and avoided detection as well as those who were detected, against the larger sample of youth who did not hack. The findings demonstrated key differences in the behaviors and attitudes of youth on the basis of their risk of detection, particularly regarding their access to technology and levels of parental supervision. The implications of this analysis for our understanding of ways to deter early onset hacking, and hacker behavior more generally, were discussed in detail.

## Understanding Computer Hacking and Hacker Behaviors

Social science research over the last few decades have revealed hacking to be a skill set that can be applied for both malicious and/or legitimate purposes (Holt, 2007, 2010; Holt et al., 2010; Steinmetz, 2015, 2017). The concept of hacking emerged in the 1950s at the Massachusetts Institute of Technology as a way to reference the manipulation of technology to produce an outcome that was different form its intended use (Levy, 1984). Hacking as a form of non-deviant manipulation has continued through today, including open-source software programming and computer hardware manipulation (Levy, 1984; Taylor, 1999; Coleman, 2014).

At the same time, a proportion of individuals engage in hacking for criminal applications, affecting business, citizens, and governments (Steinmetz, 2015). The rise of criminal hacking began in the late 1970s and 1980s, concurrent with the growth of personal computers and rudimentary Internet connectivity (e.g., Hollinger and Lanza-Kaduce, 1988). Juveniles became interested in technology during this period, using their expertise to hack financial systems and sensitive networks (Slatalla and Quittner, 1995; Furnell, 2002; Schell and Dodge, 2002). In fact, small groups of teenage hackers with names like the “414 gang” and the “Masters of Deception” targeted high-profile companies and infrastructure, generating significant concern over the way youth may become involved in criminal activities online (Slatalla and Quittner, 1995; Calce and Silverman, 2008; Yar, 2013).

Qualitative research has found that the onset of hacking occurs during early adolescence, similar to offline forms of anti-social and deviant behavior (Jordan and Taylor, 1998; Holt, 2007). During this period, individuals tend to engage in minor, simplistic hacks as they gain insight into computer technology and methods of hacking generally (Taylor, 1999; Holt, 2007). As one’s technical skill increases, so does the escalation of their offending frequency and severity. As a result, there is a need to understand the factors associated with the detection of hacking during this period so as to improve our comprehension of potential desistance factors that may reduce hacking over the long term (Maimon et al., 2014; Holt and Bossler, 2016; NCA, 2017).

Few studies have considered the factors that may be associated with the detection of hacking during adolescence, or during late adolescence in college samples (see Maimon et al., 2014; Holt and Bossler, 2016). Traditional criminological theories provide direction for factors that may be associated with an increased likelihood of being caught engaging in delinquent behaviors, including hacking. In fact, multiple correlates of hacking are consistent with predictors of traditional acts of crime and delinquency. To that end, Gottfredson and Hirschi’s (1990) general theory of crime has been found to predict individual involvement in hacking behaviors, such as password guessing to access accounts and alter content without permission from the owner (Bossler and Burruss, 2011; Holt et al., 2012, 2019; Marcum et al., 2014; Udris, 2016). Gottfredson and Hirschi (1990) argued that crime is a choice derived from weighing the costs and benefits of offending, including the risk of detection. They suggest this decision is influenced by one’s level of self-control when presented with opportunities to offend.

The level of self-control an individual has is a result of their parents’ ability to monitor, recognize, and punish deviant behavior when they occur, thereby instilling a capacity to regulate one’s actions in the moment (Gottfredson and Hirschi, 1990). Self-control is also established in early childhood, possibly accounting for early onset delinquent and anti-social behaviors (Pratt and Cullen, 2000; Vazsonyi et al., 2017). In essence, individuals with higher levels of self-control are more likely to restrain themselves when encountering criminal opportunities, while those with lower levels of self-control are more likely to take advantage of those same opportunities even when higher levels of risk detection are present. As a result, it is hypothesized that youth who hack are more likely to have low self-control compared to the general population, regardless of their risk of detection.

In much the same way, involvement in hacking is situationally dependent on access to computers and Internet connectivity. The role of opportunity as a predictor for hacking is under-examined, however, especially among juvenile populations (see Holt et al., 2019). To that end, it is virtually impossible to hack without having access to computers, mobile devices, and the Internet. Technology is readily available in most nations, creating near-constant opportunities to offend. As a consequence, criminological research demonstrates an important association between factors that increase the perceived risk and effort involved in committing an offense and reduced willingness to act on criminal opportunities (Cohen and Felson, 1979; Felson, 1986, 1995; Reyns, 2010). Resources that increase behavioral monitoring and create opportunities to intervene in offending activities may reduce individuals’ situational willingness to offend (Reyns, 2010).

Various studies have examined opportunity factors and cybercrime offending with varying results. For one, Maimon et al. (2014) investigated the influence of a warning banner on the frequency and duration of hacking incidents directed at computer systems online (see also Wilson et al., 2015). The study found that while the use of warning banners did not lead to an immediate discontinuation of the hacking incident, it reduced the duration of each hacking incident. These findings support the proposition that increased risk of detection may decrease the offending behaviors of motivated hackers.

Since many individuals report engaging in early hacking behaviors at home (Taylor, 1999; Holt, 2007), increased monitoring of computer use or limiting the amount of time one spends on the computer may reduce opportunities to hack. Similarly, the more supervision and monitoring of computer activity, the more likely an individual’s actions will be observed and punished (Marcum et al., 2014). There may, however, be economic barriers to technology access that may affect an individual’s risk of detection for hacking. Families that only own one computer may keep it in an open place where it can be accessed by all, making its usage more easily observed by parents and/or guardians. In contrast, youth who own their own device may be able to conceal their actions from others more easily. Similarly, youth who have their own rooms may encounter lower levels of detection from parents and/or guardians (e.g., parental supervision), as technology use is harder to monitor and supervise in closed areas than in open spaces.

An additional element that may be associated with hacking and the risk of detection is youths’ relationship with their parents and/or guardians. Research has found a consistent relationship between parental bonds and delinquency, as those with weak attachments to parents are at greater risk of engaging in deviance (Hirschi, 1969; Gottfredson and Hirschi, 1990; Sampson and Laub, 2003). Further, a lack of emotional ties to one’s parents may diminish their capacity to regulate behavior, negatively impacting their capacity to form relationships with pro-social peers throughout adolescence (Wright et al., 1999; Li, 2004). Parental supervision is also an important element to detecting delinquent and anti-social behaviors in the home, as noted across multiple criminological theories (Hirschi, 1969; Gottfredson and Hirschi, 1990; Sampson and Laub, 2003). When parents are able to exert direct control over their children through behavioral monitoring and punishing anti-social behavior, they are more likely to reduce their child’s involvement in delinquency (Sampson and Laub, 2003).

The role of parental bonds with respect to hacking is particularly salient as individuals are most likely to hack while at home due to ease of access to computers, and greater uninterrupted time while using the device. Limited research revealed a significant association between strong social bonds, high self-control, and reduced risk of hacking among Korean youth (Kong and Lim, 2012; Bae, 2017). Similarly, two recent studies utilizing an international population of juveniles found a relationship between reduced parental supervision, low self-control, and self-reported hacking (Udris, 2016; Back et al., 2018). It is hypothesized that those with weaker parental attachment and lower parental supervision will be more likely to hack without being detected. In contrast, those who are detected will likely have weaker parental attachments and higher levels of supervision, increasing their risk of detection.

There are also demographic factors that may shape the risk of both involvement in hacking and the likelihood of detection. First, there is a clear gender difference in the rates of hacking reported in both quantitative and qualitative samples (Gilboa, 1996; Jordan and Taylor, 1998; Taylor, 1999; Schell and Dodge, 2002; Hutchings and Chua, 2017; Holt et al., 2019). Males report higher levels of hacking, which appears to be a result of differential access to technology between the sexes from early ages (Taylor, 1999; Hutchings and Chua, 2017). There is less research considering whether girls who hack are more likely to be detected than boys at early ages. Evidence suggests females may experience greater levels of parental supervision which reduce available opportunities to offend, even in online spaces (Daigle et al., 2007; Lanctôt and Guay, 2014). As a result, boys may be more likely to hack though there may be no gender difference with respect to the risk of detection for hacking.

A small number of studies also demonstrate that individuals who hack may be from higher socio-economic status backgrounds and larger cities due to greater access to technology (Schell and Dodge, 2002; Holt, 2007; Steinmetz, 2016). Few quantitative studies have examined this relationship (Marcum et al., 2014; Holt and Bossler, 2016), though recent research by Holt et al. (2019) found that youth in smaller cities and higher socio-economic status families were more likely to self-report hacking during adolescence. It may be that families in higher socio-economic status groups provide opportunities for technology use, which reduces their risk of detection. Similarly, youth living in smaller cities may have an increased risk of detection because of reduced opportunities for unstructured socialization, as well as greater social bonds to parents (Gardner and Shoemaker, 1989).

### The Current Study

Though our understanding of hacking has increased substantially over the last two decades, research assessing the factors that predict an individual’s involvement and detection in hacking are scant. This study tested multiple hypotheses regarding the risk of detection for computer hacking which has been largely under-examined in social sciene research to date (Maimon et al., 2014; Holt and Bossler, 2016). First, it is expected that individuals with low self-control will be more likely to hack, regardless of the likelihood of detection (e.g., Holt et al., 2012; Marcum et al., 2014; Udris, 2016; Back et al., 2018). Second, youth with greater access to and frequent use of technology in private settings will be more likely to hack without detection. Third, those who engage in piracy and spend more time with peers will be more likely to hack overall, regardless of their likelihood of detection. Fourth, youth with weaker parental attachments and supervision will be more likely to hack without being detected, though those who are detected will have no difference from the general population in terms of their level of supervision.

Fifth, socio-economic status may be associated with hacking and reduced risk of detection because of greater access to technology. Sixth, geographic location may influence the risk of detection for those living in smaller towns due to differences in parental monitoring and bonding. Finally, it is expected that males will be more likely to report hacking behaviors regardless of their risk of detection due to the gendered nature of hacking. The implications of this analysis for our understanding of the factors affecting individuals’ risk of detection, as well as effective prevention and intervention efforts to affect juvenile hacking, were discussed in detail.

### Data and Methods

To test the proposed hypotheses, this analysis utilized the Second International Self-Report of Delinquency study dataset (ISRD-2, Junger-Tas, 2010; Junger-Tas and Marshall, 2012). The respondent population of the ISRD-2 consisted of juveniles in grades 7 through 9 across 30 nations, representing North America, Latin America, and some of the EU.^1^ Probability sampling was used in classrooms nested within schools to obtain respondents in small and large cities within each country (see Marshall and Enzmann, 2012 for more detail). Surveys were administered between 2005 and 2007 in school classrooms for students to complete via pencil and paper instruments. Computerized questionnaires were administered in Denmark, Finland, and Switzerland, though the data is not different from that of the larger survey population. Additionally, the sample included students attending public, private, vocational, and technical schools to reflect the diversity of educational experiences.

Such a dataset is essential to examine the extent to which hacking behaviors are identified among those who hack, as this question has yet to be addressed in survey research to date (Holt and Bossler, 2016). Furthermore, there is generally little research cultivating international samples of youth to assess their self-reported hacking behaviors (Taylor, 1999; Holt et al., 2019). The ISRD-2 is one of the few data sets available that provides a sufficient population to identify any behavioral, attitudinal, and demographic correlates of hacking behaviors and the risk of detection for these activities.

The full dataset contained 68,507 respondents, however, the final sample used in this analysis consisted of 51,059 based on missing or incomplete data. The loss of 25% of the total population did not affect the representative nature of the sample, as the respondent population resembled the original data set with respect to gender (49.2% female and 48.8% male) and age (mean = 1.08 in both samples). Additionally, the data were relatively equal with regard to geographic distribution: 26.8% of the final sample livedd in cities with less than 100,000 residents compared to 22.4% in the overall sample.

### Dependent Variable

The dependent variable for the current study was juveniles’ self-reported involvement in hacking. Respondents were asked if they ever used a computer for “hacking,” and to specify if “the last time you did it were you found out?” A relatively small proportion of respondents reported engaging in hacking behaviors at any time (*N* = 3,733; 7.3%), and only 25.2% of those individuals (*N* = 943) were detected (see Table 1). Though the overall rate of self-reported hacking is relatively low, it is consistent with prior rates reported among youth (Holt et al., 2012; Marcum et al., 2014) and late adolescent populations (Skinner and Fream, 1997; Rogers et al., 2006; Bossler and Burruss, 2011; Holt et al., 2010). The relatively small number of individuals who reported being detected for hacking allowed for the construction of a three-item variable: those who did not hack (0), those who hacked and were not discovered (1), and those who hacked and were caught (2). This measure enabled a comparison between those who did not report hacking against the other two categories which reflected 5.5% and 1.8% of the sample respectively.

**TABLE 1:** Descriptive Statistics (*N* = 51,059), Clustered by School (*N* = 1,183).

Variables	Description	N	Mean	SD	Min.	Max.
***Dependent Variables***						
Hacking Behavior			0.092	0.346	0	2
	0 = Did not hack	27,325				
	1 = Hacked/not detected	2,790				
	2 = Hacked/detected	943				
***Opportunity Characteristics***						
Own Computer			0.854	0.351	0	1
	0 = No	7,478				
	1 = Yes	43,581				
Own Mobile			0.896	0.303	0	1
	0 = No	5,310				
	1 = Yes	45,749				
Own Room			0.754	0.429	0	1
	0 = No	12,535				
	1 = Yes	38,524				
***Engagement Characteristics***						
Technology Use			4.184	1.363	1	6
	1 = None	1,358				
	2 = 1/2 h	4,270				
	3 = 1 h	10,993				
	4 = 2 h	13,236				
	5 = 3 h	9,401				
	6 = 4 h +	11,801				
Piracy			0.490	0.499	0	1
	0 = No	26,042				
	1 = Yes	25,017				
***Contextual Characteristics***						
Self-Control	12-item additive index, α = 0.83		60.674	20.252	0	100
Family Bond	4-item additive index, α = 0.55		80.636	17.0566	0	100
Time Peers			4.212	1.653	1	6
	1 = None	5,011				
	2 = 1/2 h	3,893				
	3 = 1 h	7,651				
	4 = 2 h	9,451				
	5 = 3 h	8,793				
	6 = 4 h +	16,260				
Parental Supervision			2.556	0.590	1	3
	1 = Never	2,622				
	2 = Sometimes	17,428				
	3 = Always	31,009				
***Demographic Characteristics***						
Age			1.089	0.272	0	3
	0 = Less than 12	49				
	1 = 12 to 15	47,161				
	2 = 16–17	3,655				
	3 = 18 and older	102				
Gender			0.489	0.499	0	1
	0 = Female	26,111				
	1 = Male	24,948				
Car Ownership			0.874	0.330	0	1
	0 = No	6,451				
	1 = Yes	44,608				
Small City			0.268	0.443	0	1
	0 = Larger than 100,000	37,375				
	1 = Smaller than 100,000	13,684				

It is important to note that the measure used in this survey did not define what constitutes hacking, which is different from the broader quantitative literature on hacking (Bossler and Burruss, 2011; Holt et al., 2012; Marcum et al., 2014). This measure does not enable an assessment of specific factors unique to any form of hacking that may have increased the risk of detection, such as the target of the offense or the technical skills needed to complete the activity (Holt, 2007; Steinmetz, 2016). At the same time, the use of a more general measure allowed respondents to identify what they considered as a hack without any value judgments as to whether the hack was legitimate or unethical (Holt, 2007; Steinmetz, 2016). This sort of measure may be more reflective of the diverse range of behaviors associated with hacking, including both minor and serious activities as well as those with ethical and malicious applications (Jordan and Taylor, 1998; Taylor, 1999; Holt, 2007; Steinmetz, 2016).

### Independent Variables

To assess opportunities to use technology, two binary variables were created from the following items: 1) “Do you have a computer at home that you are allowed to use?” (*own computer*) and 2) “Do you own a mobile phone?” (*own mobile*). A third opportunity measure was included to assess the impact of having a personal space where an individual may be able to utilize a computer: “do you have a room of your own?” (*own room*: 0 = no; 1 = yes).

A set of two measures were included to examine the relationship technology use and online activities. One item assessed: 1) “Outside school how much time do you spend on an average school day on each of these activities: watching tv, playing games, or chatting on the computer?” using a six-item response: (*tech use*: 1 = “none”; 2 = “30 min”; 3 = “one hour”; 4 = “two hours”; 5 = “three hours”; 6 = “four hours plus”). The second item captured individual’s self-reported digital piracy through responses to the following question: “when you use a computer did you ever download music or films during the last 12 months?” (*piracy*: 0 = no; 1 = yes).

To measure *self-control*, a variable was created using responses to 12 of the original 24-item index created by Grasmick et al. (1993). The measures capture four of the six dimensions of self-control (i.e., impulsivity, risk-taking, volatile temperament, and self-centeredness), which has been validated through prior research (Marshall and Enzmann, 2012; Rocque et al., 2013; Botchkovar et al., 2015). The Percentage of Maximum Possible (POMP) scoring method was used to create the measure for this analysis by first rescaling the 12-item measures from 0 to 100 to create an average score for each respondent (alpha = 0.83). Lower individual scores reflected lower levels of self-control.

In order to assess the relationship between time spent with peers, hacking, and the likelihood of detection, a six-item measure was created based on responses to the question: “Outside school how much time do you spend on an average school day.hanging out with friends” (*time peers*: 1 = “none”; 2 = “30 min”; 3 = “one hour”; 4 = “two hours”; 5 = “three hours”; 6 = “four hours plus”). It is hypothesized that increased time spent with peers should increase opportunities to offend, whether on or off-line (Osgood et al., 1996; Haynie and Osgood, 2005; Hoeben et al., 2016).

To measure family bonding, a mean score was created from the following four items: (1) “how do you usually get along with the man you live with (father, stepfather…)”; (2) “how do you usually get along with the woman you live with (your mother or stepmother)?”; (3) “how often do you and your parents (or the adults you live with) do something together, such as going to the movies, going or a walk or hike, visiting relatives, attending a sporting event, and things like that?”; and (4) “how many days a week do you usually eat the evening meal with (one of) your parents (or the adults you live with)?” Responses for each item were summed and then transformed into a POMP measure (Cohen et al., 1999), with higher scores indicating greater presence of the measure. The use of this POMP family bonding scale is common in studies utilizing the ISRD-2 (Botchkovar et al., 2015; Posick and Rocque, 2015) to produce a reliable family bonding measure (alpha = 0.55). An additional measure of parental supervision of general behavior was also included, asking respondents: “Do your parents (or the adults you live with) usually know who you are with when you go out?” A three-item response was provided (*parsup*: 1 = “never”; 2 = “sometimes”; 3 = “always/I don’t go out”).

To examine the hypotheses related to demographic factors and hacking, a set of four measures were used in this analysis. A four-item measure for *age* was included (0 = “less than 12”; 1 = “12 to 15”; 2 = “16–17”; 3 = “18 and older”), along with a binary measure for family *car ownership* (0 = no; 1 = yes) as a proxy for both socio-economic status (see also Holt et al., 2019). A binary measure was included to capture whether the respondent lived in an important city within their country, or a large city or town of more than 100,000 residents, or one with less than 100,000 or not considered important relative to their nation (*small city*: 0 = more than 100,000; 1 = less than 100,000). Lastly, a binary measure was created for gender (0 = female; 1 = male) to examine its relationship to self-reported hacking and likelihood of detection (Bachmann, 2010; Gilboa, 1996; Hutchings and Chua, 2017).

### Findings

To assess the behavioral and attitudinal factors associated with hacking and the risk of detection, a multinomial regression model was estimated (see Table 2). Respondents who did not self-report involvement in hacking within the last year served as the reference category, compared to those who hacked without detection, and those who hacked and were caught. The large number of respondents across the various countries sampled created unique variations within and across the study populations. The regressions were estimated using the cluster command by school (*N* = 1,183) using STATA-13 statistical software to reduce the size of both the intra-cluster correlations and standard errors. No evidence of multicollinearity could be found between the variables in the models, as no VIF was higher than 1.22, while no tolerance was lower than 0.81. The findings demonstrated key differences between these populations. First, those who hacked without detection were more likely to have their own computer and mobile device than those who did not hack. Additionally, they were more likely to spend greater amounts of time on a computer or television, as well as spend more time with peers. These access factors likely increased individuals’ opportunities to engage in online deviance. Additionally, those who hacked without detection were more likely to have engaged in piracy over the last year.

**TABLE 2:** Multinomial Regression Model for Hacking and Detection (*N* = 51,059), Clustered by School (*N* = 1,183).

	Hacked/Not Detected		Hacked/Detected	
Variables	Coef.	SE	Coef.	SE
***Opportunity Characteristics***				
Own Computer (1 = Yes)	0.335***	0.084	0.158	0.129
Own Mobile (1 = Yes)	0.211*	0.087	–0.021	0.128
Own Room (1 = Yes)	0.082	0.053	0.100	0.086
***Engagement Characteristics***				
Technology Use	0.144***	0.018	0.027	0.028
Piracy (1 = Yes)	1.618***	0.058	1.508***	0.090
***Contextual Characteristics***				
Self-Control	−0.016***	0.001	−0.013***	0.002
Family Bond	−0.006***	0.001	−0.007***	0.002
Time Peers	0.045**	0.014	0.063**	0.023
Parental Supervision	−0.293***	0.034	–0.019	0.059
***Demographic Characteristics***				
Age	0.065	0.036	0.007	0.078
Gender (1 = Male)	1.158***	0.047	0.823***	0.073
Car Ownership	0.181*	0.076	0.209	0.127
Small City	0.076	0.046	0.657***	0.067

F = 122.60***; *p < 0.05, **p < 0.01, ***p < 0.001. ^1^This study was conducted in 15 western European countries (Austria, Belgium, Denmark, Finland, France, Germany, Ireland, Italy, The Netherlands, Portugal, Spain, Sweden, Iceland, Norway, and Switzerland), 10 eastern European countries (Cyprus, the Czech Republic, Estonia, Hungary, Lithuania, Poland, Slovenia, Armenia, Bosnia-Herzegovina, and Russia), the United States (Illinois, Massachusetts, New Hampshire and Texas), and several countries outside of Europe and North America (Aruba, Netherlands Antilles, Suriname, and Venezuela).

Those who hacked without detection were also more likely to have lower levels of self-control, lower parental supervision, and lower bonds to family. These conditions likely increased individuals’ willingness to engage in wrongdoing and decreased their perceived risk of detection.

Individuals who hacked without detection were also more likely to be male and have a family car. Age group was approaching significance (0.065), with individuals in higher age groups demonstrating a greater likelihood of hacking. Living in a small town was not significant in the model, suggesting no geographic difference between the two groups.

The use of technology was not significantly different between those who hacked and were detected and those who did not hack. The only difference between these two groups with respect to opportunity variables were that they were more likely to spend time with peers and engage in piracy.

Additionally, those who were detected had lower levels of self-control and weaker family bonds compared to those who did not hack. The fact that parental supervision was non-significant, as were the technology use variables, suggests that those who hacked may have acted on opportunities to offend but were more likely to be observed compared to those who hacked without detection.

Lastly, individuals who were detected were more likely to be male and live in smaller towns. This relationship reflects both the observed gender differences in hacking, as well as potential differences in the likelihood of detection for individuals who reside in smaller geographic areas.

## Discussion and Conclusion

Research examining juvenile delinquency highlights the need to deter future wrongdoing through detection and punishment of behavior (Nagin and Pogarsky, 2001; Pratt et al., 2006). The growth of the Internet and computer technology have created new platforms to engage in delinquent acts, many of which are difficult to observe in real time compared to traditional offline delinquency (Maimon et al., 2014; Marcum et al., 2014; Holt and Bossler, 2016). As a result, there is a need to consider the factors associated with the likelihood of detection for online offending among juveniles in order to develop better prevention and treatment programs (Holt and Bossler, 2016; NCA, 2017). This study attempted to address this issue through an examination of the behavioral and attitudinal correlates of juveniles’ self-reported involvement in computer hacking and whether their behaviors were detected. A multinomial regression model was estimated using an international sample of juveniles collected through the ISRD-2 dataset (Junger-Tas and Marshall, 2012).

The findings demonstrated key support for all of the hypothesized relationships identified within the extant literature. First, low self-control was a significant predictor of hacking, regardless of whether the individual’s behavior was detected. This finding is consistent with the broader hacking literature that show individuals with low self-control to be significantly more likely to engage in various hacking behaviors (Bossler and Burruss, 2011; Holt et al., 2012, 2019; Marcum et al., 2014; Udris, 2016). In fact, youth with low self-control were more likely to act on opportunities to hack, even in the face of detection from formal and informal sources of control as a result of their volatile temperament, impulsivity, self-centeredness, and risk-taking nature (Gottfredson and Hirschi, 1990; Bossler and Burruss, 2011).

This analysis also found partial support for opportunity factors and the risk of detection related to hacking. While having access to one’s own computer and mobile phone were significantly related to hacking undetected, having a private bedroom was non-significant in both models. As a result, having one’s own device may be a bigger factor in reducing the risk of detection compared to having a private physical space in which to operate (Jordan and Taylor, 1998; Holt, 2007; Steinmetz, 2016). If individuals must utilize a shared computer, it may increase the risk of detection due to the introduction of new programs or hardware and software that may be needed in order to hack. This is reinforced by the fact that there were no differences in technology ownership and use behaviors between those whose hacking behaviors were detected and those who did not hack. In much the same way, respondents who reported engaging in piracy were significantly more likely to hack, regardless of whether their activities were identified (Holt and Copes, 2010; Bossler and Burruss, 2011; Holt et al., 2012). Thus, greater access to and use of technology may decrease an individual’s risk of detection for hacking generally.

In addition, time spent with peers was a significant predictor of hacking behavior, regardless of the likelihood of detection. The significant influence of delinquent peers on individual offending has been consistently identified in research on delinquency online (Bossler and Burruss, 2011; Holt et al., 2012; Marcum et al., 2014) and offline (Osgood et al., 1996; Haynie and Osgood, 2005; Hoeben et al., 2016). In fact, spending time with friends can provide models for offending and justifications for delinquency that increase an individual’s risk of offending generally. This finding is compounded by the significant relationship identified between diminished parental supervision and undetected hacking. If parents do not know who their child spends time with, they may be more likely to socialize with delinquent peers (Hirschi, 1969; Sampson and Laub, 2003; Posick and Rocque, 2015).

The role of weakened family bonds and diminished supervision was also significantly associated with hacking without detection. This finding is consistent with previous research as those with weak parental attachments were at greater risk of engaging in deviance (Hirschi, 1969; Gottfredson and Hirschi, 1990; Wright et al., 1999; Sampson and Laub, 2003; Li, 2004; Posick and Rocque, 2015; Udris, 2016; Back et al., 2018). The role of parental bonds with respect to hacking is particularly salient as youth seem most likely to hack while at home due to ease of access to computers and greater uninterrupted time while using the device. The absence of significant differences between those who did not hack and those whose hacks were detected suggests the need for parental attachments and youth involvement in order to decrease the risk of juvenile hacking, similar to traditional delinquency.

The study also found several demographic factors associated with hacking. Those whose families owned a car were more likely to hack undetected, which may be a proxy for differential opportunities to use technology as a function of economic advantage (Schell and Dodge, 2002; Holt, 2007; Steinmetz, 2016; Holt et al., 2019). Males were also more likely to hack, whether detected or undetected, consistent with both previous quantitative and qualitative studies on hacking behaviors (Gilboa, 1996; Jordan and Taylor, 1998; Taylor, 1999; Schell and Dodge, 2002; Hutchings and Chua, 2017; Holt et al., 2019). It is unclear if this dynamic reflects differential supervision of behavior based on gender (Daigle et al., 2007; Lanctôt and Guay, 2014), or more unique factors associated with computer hacking generally. Lastly, youth living in smaller cities were more likely to have their hacking detected. This may be a function of reduced opportunities for unstructured socialization, as well as greater social bonds to parents as identified in prior research (Gardner and Shoemaker, 1989). These dynamics require further research in order to understand the role of demographic factors in the risk of online offending generally (Hutchings and Chua, 2017; Holt et al., 2019).

This study has direct implications for the development of programs to reduce juvenile hacking, as few have considered the factors that may increase the potential for obfuscation or detection of computer hacking (Holt and Bossler, 2016; NCA, 2017). The findings from the multinomial regression models demonstrated that hacking has some unique qualities that differentiate it from offline offending (see Bossler and Burruss, 2011; Steinmetz, 2016), but shared behavioral and attitudinal factors similar to that of traditional delinquency. As a result, there may be no need for specialized delinquency prevention programs for cybercrime. Instead, practitioners may benefit from incorporating information regarding simple forms of computer hacking into existing programmatic materials. Additionally, there is a need to increase parental awareness of cybercrime as a form of juvenile delinquency so as to improve the degree of supervision and oversight that may reduce opportunities to hack (Holt et al., 2012; Marcum et al., 2014). Lastly, substantive empirical research is needed to develop and evaluate the success of any prevention program that may emerge, whether in traditional delinquency reduction programs or those unique to cybercrime generally (Holt and Bossler, 2016; Leukfeldt, 2017; NCA, 2017).

Though this study provides an examination of an under-studied issue associated with juvenile hacking, there are several limitations that must be noted. First, these data were collected between 2005 and 2007 when both the Internet and computer technology were less advanced and more costly. Future research would benefit from exploring whether the significant relationships identified in this analysis are also present in a more contemporary sample of youth. Relatedly, the current study is limited by its use of a predominately Western sample population. Future research should explore whether these factors are differentially associated with hacking and detection among Asian, Oceanic, African, and other nationally representative populations (Holt and Bossler, 2016).

The cross-sectional design of this study also presents some limitations as to the theoretical implications of this analysis. Cross-sectional studies provide important information regarding significant relationships between concepts and variables, though longitudinal data is needed to advance understanding of the temporal causes, pathways, and trends of juvenile hacking and detection (Holt et al., 2012; Marcum et al., 2014; Udris, 2016; NCA, 2017). The secondary nature of the data also limited the potential to examine the nature of the hacks reported by respondents, or their technical skills. It may be that individuals who engaged in more sophisticated or ethical hacks were able to continue without detection or sanction from formal and/or informal sources of social control. Furthermore, the dataset contained no measures regarding peer hacking behaviors, restricting the current study’s operationalization of peer association (Bossler and Burruss, 2011; Holt et al., 2012; Marcum et al., 2014). Such information is essential in improving our understanding of the nature of hacking and its similarities to traditional offline delinquency.

## Data Availability Statement

The Second International Self-Report of Delinquency (ISRD-2) dataset is in the ICPSR repository.

## Ethics Statement

Ethical review and approval was not required for the study on human participants in accordance with the local legislation and institutional requirements. Written informed consent to participate in this study was provided by the participants’ legal guardian/next of kin.

## Author Contributions

JL developed the primary idea, and wrote the introduction and literature review. TH conducted the statistics, wrote the methods and findings. Both wrote the discussion and conclusions and provided final revisions.

## Conflict of Interest

The authors declare that the research was conducted in the absence of any commercial or financial relationships that could be construed as a potential conflict of interest.
